# Effects of trace minerals source in the broiler breeder diet and eggshell translucency on embryonic development of the offspring

**DOI:** 10.1016/j.psj.2022.102455

**Published:** 2022-12-30

**Authors:** Henry van den Brand, Timo Hubers, Ilona van den Anker, Cibele A. Torres, Emily Frehen, Monique Ooms, Joop Arts, Bjorge F.A. Laurenssen, Marcel J.W. Heetkamp, Bas Kemp, Roos Molenaar

**Affiliations:** ⁎ Adaptation Physiology Group, Wageningen University and Research, Wageningen, the Netherlands; † Zinpro Animal Nutrition Inc., Boxmeer, the Netherlands; ‡ ABZ Diervoeding, Nijkerk, the Netherlands

**Keywords:** broiler breeder, organic trace mineral, egg translucency, chicken quality, tibia characteristics

## Abstract

In 2 experiments, interactions between trace mineral (Zn, Mn, Cu, Se) source (organic or inorganic) in the broiler breeder diet and egg translucency (high or low) on egg characteristics and embryonic development were investigated. In the first experiment, eggs from old breeders (55–57 wk) and in the second experiment, eggs from prime breeders (34–36 wk) were used. Egg composition and bacterial load on the eggshell were analyzed in fresh eggs. During incubation, metabolic heat production of the embryos (d 8 (**E8**) to 19 of incubation) and tibia ossification (E8.5–E14.5) were determined daily. At hatch, chicken quality was assessed, including tibia biophysical characteristic. Egg quality was not affected by breeder trace minerals source or egg translucency in both experiments. In both experiments, an interaction between trace minerals source and translucency score was found for egg weight loss during incubation. In inorganic trace minerals fed breeders, a high egg translucency resulted in a higher egg weight loss than a low egg translucency, whereas this difference was not seen in organic trace minerals fed breeders. Embryonic heat production and tibia ossification were not affected by trace minerals source or egg translucency. Chicken quality showed ambiguous results between experiment 1 and 2 regarding trace minerals source in the breeder diet. In experiment 2, high translucent eggs from organic fed breeders hatched later than eggs from the other three treatment groups and additionally, high egg translucency resulted in lower residual yolk weight and higher heart and liver percentage of YFBM compared to low egg translucency. Tibia biophysical characteristics at hatch were not affected by trace minerals source or egg translucency. It can be concluded that organic trace minerals source in broiler breeder diet affects eggshell conductance, particularly in low translucent eggs, but effects on chicken quality and tibia characteristics appears to be limited.

## INTRODUCTION

The eggshell is an important component of the egg having several functions, such as being a barrier against pathogen infiltration, prevention of excessive water loss during incubation and providing calcium for the embryo (Lavelin et al., 2000). Eggshell quality is affected by animal-related and environmental factors, such as breed, breeder age, diet, and ambient temperature (Rossi et al., 2013). Eggshell quality is often expressed by specific gravity, eggshell thickness, or breaking strength (Roque and Soares, 1994). One eggshell characteristic that is less considered in this perspective is eggshell translucency or eggshell transmittance or mottling. Eggshell translucency was first reported by Holst et al. (1932), but not much research has been performed to this phenomenon in later years. Recently, Wang et al. (2017, 2019a) and Zhang et al. (2021) investigated effects of egg translucency in relationship to other egg characteristics. The extent of egg translucency has been related to the amount of moisture in the eggshell or eggshell membranes (Wang et al., 2019a). Additionally, Olkowski et al. (2015) showed that eggs with different translucency scores (high vs. low) differed in shell matrix fiber architecture. A high egg translucency might be the result of irregular mammillary knobs, which might arise from fusion of several mammillary cones during early shell formation (Bain et al., 2006). Consequently, the risk of microcracks is increased in high translucent eggs. The latter might explain why Chousalkar et al. (2010) found that high translucent eggs were more penetrated by *Salmonella* and *E. coli* than low translucent eggs. However, Ray et al. (2015) showed that high translucent eggs had more externally branched pores in the eggshell than low translucent eggs, but pore structure, number of pores and eggshell thickness could not be related to penetration by *Salmonella*. Furthermore, Roberts et al., 2013 showed only low correlations between egg translucency and ultrastructural features of the mammillary layer. The evidence provided might implies that egg translucency could be an important egg quality parameter affecting hatchability and offspring quality.

Given these potential negative effects of high translucent eggs, it can be valuable to reduce the number of eggs with high eggshell translucency. One potential way is the use of trace minerals, particularly Zn, Mn, Cu, and Se, in broiler breeder diets. Trace minerals can affect the ultrastructure of the eggshell (Qiu et al., 2020) by affecting the palisade layer and the mammillary cone width (Stefanello et al., 2014; Li et al., 2018, 2019; Qui et al., 2020). Consequently, trace minerals, besides Ca and P, might play a role in eggshell quality characteristics (Araújo et al., 2019; Qui et al., 2020). Because of the role of trace minerals on the mammillary layer of the eggshell, a potential effect on the eggshell translucency can also be hypothesized. Additionally, specific trace minerals (Zn, Mn, Cu) play a role in eggshell or eggshell membrane formation via carbonic anhydrase (Zn; Zhang et al., 2017; Li et al., 2019), glycosaminoglycan (Mn; Xiao et al., 2014) or lysyl oxidase (Cu; Akagawa et al., 1999). Altogether, it can be hypothesized that trace minerals can affect shell or membrane characteristics of eggs and consequently affect egg translucency. Additionally, it can be hypothesized that trace mineral source might particularly of influence in older layers or breeders, because in general older layers or breeders have poorer eggshell quality than younger layers or breeders (for review see Nasri et al., 2020).

Trace minerals are currently included in poultry diets for breeders and broiler chickens mostly as inorganic salts (Sirri et al. 2016). However, research in the last decades has demonstrated that trace minerals bound to amino acids or proteins, called organic trace minerals, have a higher bioavailability than inorganic trace minerals (Wedekind et al., 1992; Star et al., 2012). Consequently, organic trace minerals have been shown to positively affect different physiological systems compared to inorganic trace minerals, not only in broiler chickens (Star et al., 2012; Güz et al., 2019; Venkata et al., 2020), but also in laying hens (Swiatkiewicz and Koreleski, 2008; Zhang et al., 2017; Li et al., 2019; Qui et al., 2020) and broiler breeders (Wang et al., 2019b; Yaqoob et al., 2020; Güz et al., 2021). Moreover, also transgenerational effects on the offspring during the embryonic phase (Favero et al., 2013a; Xiao et al., 2015; Zhu et al., 2015) or later life rearing (Kidd et al., 1992; Hudson et al., 2004a; Virden et al., 2003, 2004; Araújo et al., 2019) have been found. Physiological systems positively affected by the use of organic trace minerals instead of inorganic trace minerals are among others the reproductive system (Hudson et al., 2004b; Favero et al., 2013b; Araújo et al., 2019; Wang et al., 2019b; Yaqoob et al., 2020), eggshell formation and quality (Mabe et al., 2003; Swiatkiewicz and Koreleski, 2008; Xiao et al., 2014; Zhang et al., 2017; Li et al., 2019; Qui et al., 2020), embryonic development (Favero et al., 2013b; Zhu et al., 2015), bone development (Swiatkiewicz and Koreleski, 2008; Favero et al., 2013a; Venkata et al., 2020), immune system (Hudson et al., 2004b; Troche et al., 2015), and antioxidative system (Zhu et al., 2015; Wang et al., 2019b; Venkata et al., 2020; Yaqoob et al., 2020).

It can be hypothesized that organic trace minerals fed to broiler breeders will be able to affect egg translucency, embryonic development, and hatchling quality. The aim of this study was to investigate the interaction between trace minerals source in the broiler breeder diet and egg translucency on embryonic development and hatchling quality.

## MATERIALS AND METHODS

### Experimental Design

In 2 experiments, effects of trace minerals (Zn, Mn, Cu, Se) source in broiler breeder diets and egg translucency on egg characteristics and embryonic development of the offspring were investigated. Both experiments had a 2 × 2 factorial arrangement with trace minerals source (inorganic vs. organic) and egg translucency (high vs. low) as factors, resulting in 4 treatment groups. Each experiment consisted of 2 consecutive batches, each containing the 4 treatments. Measurements were performed before and during incubation and after hatching on the offspring. All experimental protocols were approved by the Central Commission on Experimental Animals (the Hague, the Netherlands), approval number AVD1040020198144.

### Experimental Animals and Diets

In both experiments, eggs were obtained from commercial Ross 308 broiler breeder farms (1 farm per experiment). At each farm, breeders were housed in one of 2 identical breeder houses. Breeders in one house were fed a control diet with inorganic trace minerals Zn, Mn, Cu and Se, whereas breeders in the other house were fed a diet in which part of the inorganic trace minerals Zn, Mn, Cu and Se were replaced by their organic varieties (ZMC-Se, Zinpro Animal Nutrition Inc., the Netherlands). Breeder age was 55 to 57 wk and 34 to 36 wk at moment of egg collection for the 2 batches in experiment 1 and 2, respectively. Diet composition for both treatments per breeder farm was the same, except for Zn, Mn, Cu, and Se (Table 1). Diets were produced by ABZ Diervoeding (Eindhoven, the Netherlands) and fed as meal. Diets were analysed on CP, crude fat, crude fiber, ash, Zn, Mn, Cu, Se, Fe, Na, Ca, and P by a commercial lab (Eurofins, Rotterdam, the Netherlands; Table 1). Diets were fed for at least 9 wk before eggs were collected.

**Table 1:** Diet composition and calculated and analysed nutrient content of broiler breeder diets, containing either inorganic or organic trace minerals (Zn, Mn, Cu and Se), and fed to two broiler breeder flocks (aged 55 to 57 wk; experiment 1 or aged 34 to 36 wk; experiment 2) (g/kg as-fed).

	Experiment 1		Experiment 2	
	Inorganic	Organic	Inorganic	Organic
Ingredient				
Oat	10.0	10.0		
Maize, rolled	425.5	425.5	320.0	320.0
Maize meal	20.0	20.0	20.0	20.0
Wheat rolled	216.6	216.7	251.8	251.8
Wheat gluten feed meal			60.0	60.0
Rapeseed expeller			29.4	29.4
Rapeseed, extracted	79.2	79.2	30.0	30.0
Soya, extracted 49%	45.8	47.5	53.7	52.9
Soya, extracted Hipro 48/3.5			15.0	15.0
Sunflower seed expeller, 29% CP			26.7	22.2
Sunflower seed expeller 38% CP	76.3	73.9	52.5	57.0
Peas			15.0	15.0
Citrus peel liquor	14.9	14.9	9.9	9.9
Palm oil			3.0	3.0
Salmon oil			4.0	4.0
Lecithin mix	16.2	16.2	17.9	17.9
Acid mixture	5.3	5.3	5.5	5.5
Limestone	74.0	74.7	68.9	68.9
Monocalcium phosphate	0.6	0.6	0.5	0.5
Sodium bicarbonate	1.8	1.8	1.9	1.9
Salt	1.1	1.2	1.0	1.0
Methionine (DL, 99%)	1.1	1.1	1.5	1.5
L-Lysine HCl	0.9	0.9	0.9	0.9
L-Threonine (98%)	0.5	0.5	0.7	0.7
Choline chloride			0.2	0.2
Premix A (inorganic)^1^	4.0		4.0	
Premix B (organic)^2^		4.0		4.0
Water	6.1	6.1	6.1	6.1
Total	1,000.0	1,000.0	1,000.0	1,000.0
Calculated nutrients				
ME (kcal/kg)	2,782	2,782	2,775	2,775
Crude protein (g/kg)	141.1	141.2	148.8	149.0
Crude fat (g/kg)	40.0	40.0	50.0	50.0
Crude fibre (g/kg)	40.7	40.7	45.4	45.4
Ash (g/kg)	103.5	103.4	100.0	100.0
dig. Lysine (g/kg)	5.3	5.3	5.7	5.7
dig. Methionine (g/kg)	3.39	3.39	3.80	3.80
dig. Meth. + Cyst (g/kg)	5.51	5.50	5.97	5.97
Zn (mg/kg)	113	113	115	115
Mn (mg/kg)	133	133	136	136
Cu (mg/kg)	22	22	23	23
Se (mg/kg)	0.3	0.3	0.3	0.3
Fe (mg/kg)	101	101	101	101
Na (g/kg)	1.4	1.4	1.4	1.4
Ca (g/kg)	31	31	32	32.4
P (g/kg)	4.2	4.2	4.4	4.4
Analysed nutrients				
Crude protein (g/kg)	146.0	141.5	141.3	143.0
Crude fat (g/kg)	37.5	51.0	44.0	48.0
Crude fiber (g/kg)	44.0	48.0	41.0	49.0
Ash (g/kg)	113.0	113.0	98.0	102.0
Zn (mg/kg)	100	78	83	97
Mn (mg/kg)	110	84	85	94
Cu (mg/kg)	19	15	18	16
Se (mg/kg)	0.6	1.3	0.7	0.7
Fe (mg/kg)	250	190	160	210
Na (g/kg)	1.3	0.98	0.80	0.92
Ca (g/kg)	36	29	30	33
P (g/kg)	4.3	4.5	3.9	4.3

1 Added trace minerals and vitamins per kg of diet: Fe. 100 mg; I. 2 mg; Cu (as copper sulphate pentahydrate). 15 mg; Mn (as manganese II oxide). 100 mg; Zn (as zinc sulphate monohydrate). 80 mg; Se (as sodium selenite). 0.30 mg; vitamin A. 10,000 IU; vitamin D3. 1,500 IU; vitamin D 25-hydroxy cholecalciferol. 1.500 IU; vitamin E. 100 mg; vitamin K3. 3 mg; vitamin B1. 3 mg; riboflavin. 10 mg; niacinamide. 30 mg; pantothenic acid. 16 mg; pyridoxin. 4 mg; cyanocobalamin. 30 µg; biotin. 300 µg; choline chloride. 345 mg; choline. 300 mg; folic acid. 2 mg; β-carotene. 3 mg.

2 Added trace minerals and vitamins per kg of diet: Fe. 100 mg; I. 2 mg; Cu (as copper sulphate pentahydrate). 8 mg; Cu (as copper-amino acid chelate). 7 mg; Mn (as manganese II oxide). 60 mg; Mn (as manganese-amino acid chelate). 40 mg; Zn (as zinc sulphate monohydrate) 30 mg; Zn (as zinc-amino acid chelate). 50 mg; Se (as sodium selenite) 0.10 mg; Se (as zinc-L-selenomethionine). 0.20 mg; vitamin A. 10,000 IU; vitamin D3. 1,500 IU; vitamin D 25-hydroxy cholecalciferol. 1,500 IU; vitamin E. 100 mg; vitamin K3. 3 mg; vitamin B1. 3 mg; riboflavin. 10 mg; niacinamide. 30 mg; pantothenic acid. 16 mg; pyridoxin. 4 mg; cyanocobalamin. 30 µg; biotin. 300 µg; choline chloride. 345 mg; choline. 300 mg; folic acid. 2 mg; β-carotene. 3 mg.

### Eggshell Translucency

**Figure 1:**
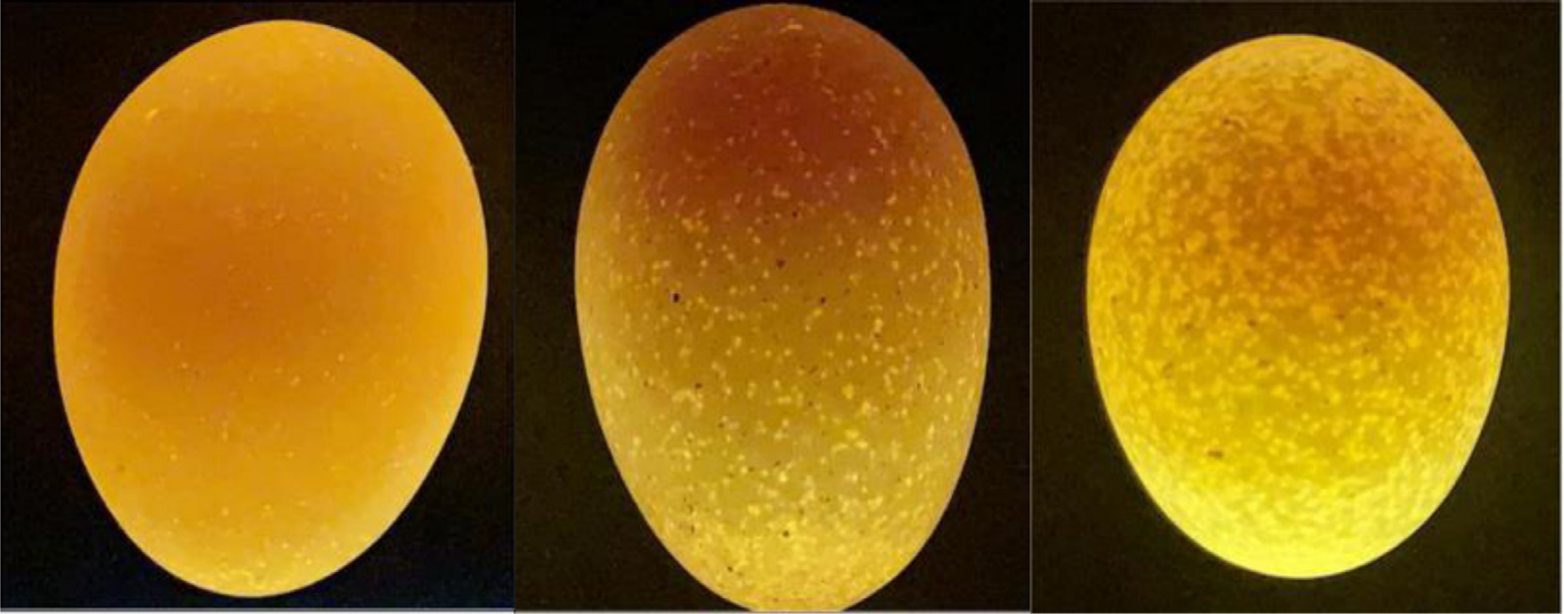
Examples of eggs with translucency scores 1 (<5% of surface has translucency spots; left), 3 (15–30% of surface has translucency spots; middle) and 5 (>40% of surface has translucency spots; right).

In both experiments, eggs were collected twice per day (10.00 and 17.00 h). Eggs of 1 collection day (both collection moments) per batch were stored at the breeder farm for 5 d at a temperature of 17 to 18°C and a RH between 60 and 70%. Per breeder diet, first-grade eggs (visual clean, no cracks) were selected on egg weight between 65.0 and 67.9 g (experiment 1) or between 60.0 and 62.9 g (experiment 2). Selected eggs were placed in a candling box and visually scored by the same person (all batches in both experiments) on egg translucency, using a range of 1 to 5. Eggs were scored as 1 when no or almost no translucency spots (< 5% of the surface) were seen in the eggshell; as score 2 when 5 to 15% of the surface consisted of translucency spots; as score 3 when 15 to 30% of the surface consisted of small translucency spots; as score 4 when 30 to 40% of the surface consisted of translucency spots; and as score 5 when >40% of the surface consisted of translucency spots or when larger translucency windows were found. In Figure 1, examples of eggs with score 1, 3, and 5 are provided. In experiment 1, eggs with a translucency score of 1 plus 2 (low) and with a translucency score of 5 (high) were selected and used in the experiment. In experiment 2, limited eggs with a low translucency score were available and consequently, eggs of translucency score 1 to 3 (low) were compared with eggs having a translucency score of 5 (high). In total, approximately 1,000 to 1,300 eggs per batch per breeder diet were scored to obtain sufficient eggs of each translucency score. From these eggs, approximately 300 eggs per batch per breeder diet per translucency score were selected and thereafter transported to the research facility of Wageningen University and Research (Wageningen, the Netherlands).

### Incubation

After arrival at the research facility, eggs were placed on incubation trays (88 eggs per tray), separately for each breeder diet and translucency score, and all placed in one incubator (HatchTech, Veenendaal, the Netherlands; maximum capacity 1,408 eggs) at a temperature of 17°C for 2 d. Trays with eggs of the different treatments were equally divided over the incubator and were not fumigated before incubation. After the 2 d of storage, incubation started in the same incubator for 8 d. Eggs were warmed in 12 h from storage temperature to an eggshell temperature (**EST**) of 37.8°C. The moment that an EST of 37.8°C was reached was defined as the start of incubation. At 4 randomly chosen eggs, divided over the different treatments, a temperature sensor (NTC Thermistors, type DC95, Thermometrics, Somerset, UK) was placed at the equator of the egg, using heat conducting paste (Schaffner Holding AG, Luterbach, Switzerland) and a small piece of tape (2 × 2 cm; Tesa BV, Almere, the Netherlands). Based on the median of these 4 sensors, the EST was maintained at 37.8°C by continuously adjusting the incubator temperature. Relative humidity was maintained between 50 and 60%. After 8 d of incubation (**E**8), eggs were candled and infertile eggs or eggs containing a dead embryo were removed. Eggs containing a viable embryo were divided over 4 climate-respiration chambers (**CRC**), which were used as incubators. Two CRC contained approximately 140 eggs each (Lourens et al., 2006) and 2 CRC contained approximately 350 eggs (Verstegen et al., 1987). Per breeder diet and translucency score, eggs were placed separately in one of the 4 CRC. Per experiment and batch, treatments rotated among the CRC. Within each CRC, at 4 randomly chosen eggs a Pt-100 temperature sensor (Sensor Data BV, Rijswijk, the Netherlands) was attached at the equator of the eggs, as described above. CRC temperature was continuously adjusted to maintain the EST at 37.8°C in all 4 CRC until E19. At E19, all eggs were candled again and eggs containing a dead embryo were removed. Furthermore, the number of eggs per CRC was reduced to 17 and these eggs were allowed to hatch. Eggs containing a dead embryo at candling, that were removed at E19 or that did not hatch were opened to determine fertility or moment of mortality as described by Lourens et al. (2006). From 461 h after start of incubation onward, the number of hatched chickens was assessed each 6 h. Newly hatched chickens were marked with a colour on their head and removed from the CRC 6 h later.

### Measurements

Before selecting eggs at the breeder farms, 300 randomly chosen eggs per batch per breeder diet were individually weighed to determine egg weight distribution. Before the start of incubation (E0), and at E8 and at E19, all selected eggs were individual weighed and egg weight loss was calculated between E0 and E19.

One day after egg collection at the breeder farm, 10 eggs per treatment (trace minerals source × translucency score) per batch were analyzed for bacterial load of the eggshell. Eggs were individually placed in jars with 40 mL sterile warm (38.0°C) PBS. Jars were closed with a lid and gently rolled for 10 min on a roller mixer (Stuart, Staffordshire, UK), whereafter the egg was removed and the PBS solution was transferred to a 50 mL sterile tube. In a flow cabinet, PBS was plated, using glass beads on brain heart infusion agar plates (100 µL per plate) after 0, 10, and 100 times dilution. Additionally, the used sterile PBS was plated as well to act as negative control. Agar plates were placed in a stove (37.8°C) for 48 h, whereafter the number of CFU were counted per plate for the different dilutions. No differentiation between species of CFU was made. CFU could be counted on the plates on which the PBS was plated undiluted, so these plates were used for counting rather than the plates at which the diluted solution was plated out.

At the d E0, 10 eggs per treatment per batch were weighed and boiled for 10 min, whereafter yolk, albumen and eggshell (including membranes) were separated. Yolk weight was determined and eggshells were dried for 24 h at room temperature, whereafter they were weighed as well. Albumen weight was calculated as egg weight – yolk weight – eggshell weight. Additionally, eggshell thickness was measured with an electronic micrometre (IP54, Helios-Preisser, Grosuplje, Germany) at the top, middle and blunt end of the egg and the average of these 3 areas was calculated.

From E8.5 to E14.5, 5 eggs containing a viable embryo per treatment per batch per day were removed from the CRCs to determine tibia ossification. From E8.5 to E10.5 complete legs and from E11.5 to E14.5 only the tibia were fixated in 4% formalin/PBS for 24 h and stored in 70% ethanol thereafter until further use. Tibia was rehydrated in 4 steps (2 × 75% ethanol (1 h each), 50% ethanol (1 h), milliQ). After O/N neutralization in saturated sodium tetraborate solution, tibias were bleached in 0.45% H_2_O_2_ and 0.85% KOH for 40 min, followed by a trypsin digestion (30 mL saturated sodium tetraborate, 70 mL MQ, 1 g trypsin (Merck, Darmstadt, Germany) for 24 h. Samples were stained for 24 h in 1% KOH with 150 mg/L Alizarin red. After de-staining (30 ml saturated sodium tetraborate, 70 mL MQ, 1 g trypsin), bones were transferred to a 1% KOH-glycerine solution (3:1 for 1 d, 1:1 for 1 d, and 1:3 for 1 d). Finally, tibia was stored in 100% glycerine. After carefully removal from the glycerine, tibia were dried on tissue and placed under a microscope (Zeiss Stemi SV 11 stereomicroscope, Jena, Germany). Tibia were photographed with a 0.63× objective at different magnifications, using an Olympus DP50 camera (Tokyo, Japan), using Olympus Analysis-five software (Tokyo, Japan). For tibia at E8.5 a magnification of 1.2 × 10 was used; for tibia at E9.5 a 1.0 × 10 magnification; for tibia at E10.5 and E11.5 a 0.8 × 10 magnification and for tibia at E12.5, E13.5, E14.5 a 0.6 × 10 magnification. In all tibia, total length of the tibia and ossified length were determined. Ossified percentage was calculated as ossified length/total length × 100.

From E8 till E19, oxygen consumption and carbon dioxide production were determined continuously from the 4 CRCs. Based on these measurements, embryonic heat production was calculated per day of incubation, using the formula of Romijn and Lokhorst (1961). Heat production was expressed in mW per egg per day and corrected for day of embryonic mortality. Respiration quotient was calculated as l CO_2_/l O_2_ and calculated per day of incubation. Prior to this study, CO_2_ recovery tests were performed as a full system check of our indirect calorimetry setup. Recovery results for the 4 CRC's were 101.1, 100.9, 97.8, and 101.5%.

Six hours after the hatched chickens were coloured on their head, they were removed from the CRCs and scored on chicken quality (BW, first or second grade, presence of red beaks or red hocks, navel quality). Chickens were considered as first grade when they did not show any deformities, such as exposed brains, 4 legs, cross beak, or extruded yolk. Red beaks and red hocks were scored as present or absent, whereas navel quality was scored as 0 (clean and closed navel), 1 (small black button (<2 mm) on the navel or small string) or 2 (large black button (>2 mm)) (Molenaar et al., 2010). Thereafter, chickens were killed by cervical dislocation, opened and organ weights (heart, liver, proventiculus + gizzard, intestines, residual yolk (**RY**)) were determined. Yolk free body mass (**YFBM**) was calculated as BW – RY. From 5 chickens per treatment per batch, divided over the hatch window, tibias were collected. These were stored at −20°C till further processing. After thawing, left tibias were placed in a GE Phoenix 3D X-ray microfocus CT scanner (General Electric Company, Boston, MA) to determine tibia proximal length, lateral cortex thickness, total volume, osseous volume, pore volume, mineral content, and mineral density (see for detailed description Güz et al., 2021).

### Statistical Analyses

All statistical analyses were performed in SAS (Version 9.4, 2013, SAS Institute Inc., Cary, NC) for both experiments separately. For continuous variables, homogeneity of variance was checked for both means and residuals. Not-normal distributed variables were log transformed before analyses. For all analyses, egg or hatchling was used as the experimental unit, except for embryonic heat production, where the CRC was used as the experimental unit.

Average egg weight at the breeder farm (300 eggs per treatment per batch randomly taken) was analyzed with a MIXED procedure, using the model:

(1) Y=μ+Treatment+Batch+e,

where Y = dependent variable, µ = overall mean, Treatment = trace minerals source in the breeder diet (inorganic, organic), Batch = batch (1, 2), e = residual error.

Distribution of egg translucency score at the breeder farm was analyzed with the GLIMMIX procedure with model 1, using a multinomial distribution and a cumlogit link.

Egg composition before incubation, egg weight (loss) during incubation and tibia length, ossification length, and ossification percentage at E8.5 till E14.5 (per day) were analyzed with a MIXED procedure, using the model:

(2) Y=μ+Treatment+Translucency+Interaction+Batch+e,

Where Y = dependent variable, Treatment = trace minerals source in the breeder diet (inorganic, organic), Translucency = Egg translucency score (low, high), Interaction = Interaction between Treatment and Translucency score, Batch = batch (1, 2), e = residual error.

Fertility of eggs (all eggs) and percentage of viable embryos at E19 (as percentage of fertile eggs, excluding the eggs that were used for tibia collection between E8.5 and E14.5), were analyzed with a GLIMMIX procedure with model 2, using a binomial distribution and a logit link.

Embryonic heat production and RQ were analyzed with a repeated analysis model, using a MIXED procedure. Model 2 was extended with day of incubation, CRC nested within batch was used as the repeated subject and an autoregressive 1 covariance structure was applied.

Continue hatchling characteristics (hatch time, weight, YFBM, RY, organ weights (as percentage of YFBM) were analyzed with a MIXED procedure, using model 2, added with the observer as a fixed factor (1 to 3). Navel score was analyzed with this model as well, using a GLIMMIX procedure with multinomial distribution and a cumlogit link, whereas beak and hock scores were analyzed with the same model, using a binomial distribution and a logit link.

Results are expressed as LSmeans ± SEM, unless indicated otherwise. Multiple comparisons were performed after correction for Bonferroni. Effects were considered to be significant at *P* ≤ 0.05.

## RESULTS

### Eggs, Fertility, and Embryonic Mortality

#### Experiment 1

The average egg weight of the 300 eggs per treatment per batch determined at the breeder farm was 68.0 g for both treatments (range 56.3 to 85.3 g; *P* = 0.97 for breeder diet). Average translucency score at the breeder farm was 3.24 ± 0.03 (SE) and 3.35 ± 0.03 (SE), for the inorganic and organic trace minerals breeder diet, respectively (*P* = 0.002). Egg weight at E8 (*P* = 0.02), E19 (*P* = 0.002) and egg weight loss between E0 and E19 (*P* = 0.02) showed all an interaction between trace minerals source in the breeder diet and translucency score (Table 2). In eggs originating from organic trace minerals fed broiler breeders, no effect of translucency score was found on egg weight (loss), whereas in eggs originating from inorganic trace minerals fed broiler breeders, egg weight was lower (E8: Δ = 0.43 g; E19: Δ = 0.69 g) and egg weight loss (Δ = 0.78%) was higher in eggs with a high translucency score than in eggs with a low translucency score. Egg weight at set (E0) did not differ between trace minerals source, but was higher in eggs with a low translucency score than in eggs with a high translucency score (Δ = 0.09 g; *P* = 0.03).

**Table 2:** Effects of trace minerals (Zn, Mn, Cu, Se) source (inorganic vs. organic) in the broiler breeder diet (55 to 57 wk of age) and egg translucency score (1 + 2 vs. 5) on egg weight at set (E0), E8, E19, and egg weight loss between E0 and E19 (LSmeans ± SEM) (Experiment 1).

	Egg wt E0, g	Egg wt E8, g	Egg wt E19, g	Egg wt loss E0 – E19, %
N	1,925	1,734	1,321	1,321
Minerals source				
Inorganic	66.41	63.41	59.53	10.31
Organic	66.36	63.38	59.57	10.22
SEM	0.03	0.04	0.06	0.07
Translucency score				
1 + 2	66.43^a^	63.55	59.78	9.99
5	66.34^b^	63.24	59.33	10.54
SEM	0.03	0.04	0.06	0.08
Interaction				
Inorganic x 1 + 2	66.49	63.62^a^	59.88^a^	9.92^c^
Inorganic x 5	66.33	63.19^c^	59.19^c^	10.70^a^
Organic x 1 + 2	66.38	63.47^ab^	59.67^ab^	10.06^bc^
Organic x 5	66.34	63.29^bc^	59.47^bc^	10.38^ab^
SEM	0.04	0.05	0.08	0.10
P-values				
Minerals source	0.19	0.59	0.65	0.36
Translucency score	0.03	<0.001	<0.001	<0.001
Interaction	0.14	0.02	0.002	0.02

a,b,c LSmeans within a column and factor lacking a common superscript differ (*P* ≤ 0.05).

Fertility was 90.4% on average and was not affected by trace minerals source in the breeder diet (*P* = 0.99), translucency score (*P* = 0.60) or the interaction between these 2 (*P* = 0.96). The percentage of vital embryos at E19 (as percent of fertile eggs) was on average 91.2% and not affected by trace minerals source in the broiler breeder diet (*P* = 0.81), translucency score (*P* = 0.24) or the interaction between these 2 (*P* = 0.98). Hatchability of the eggs transferred at E19 was on average for all treatments 98.5%.

#### Experiment 2

The average egg weight of 300 eggs per treatment per batch at the breeder farm was 60.4 g (range 47.0–71.5 g) and 59.9 g (range 45.4–74.4 g), for the inorganic and organic trace minerals, respectively (*P* = 0.006). Average translucency score at the breeder farm was 4.55 + 0.02 (SE) for both treatments (*P* = 0.42). Egg weight at E0 (*P* = 0.01), E8 (*P* = 0.006), and E19 (*P* < 0.001) and egg weight loss between E0 and E19 (*P* = 0.04) all showed an interaction between trace minerals source in the breeder diet and translucency score (Table 3). Egg weight (loss) of eggs with a high translucency score did not differ between eggs from both breeder diets, whereas at a low translucency score, eggs from breeders fed inorganic trace minerals weighed more at E0 (Δ = 0.22 g), E8 (Δ = 0.31 g), and E19 (Δ = 0.65) than eggs from breeders fed organic trace minerals, whereas the opposite was found for egg weight loss between E0 and E19 (Δ = 0.58%).

**Table 3:** Effects of trace minerals (Zn, Mn, Cu, Se) source (inorganic vs organic) in the broiler breeder diet (34 to 36 wk of age) and egg translucency score (1 + 2 + 3 vs. 5) on egg weight at set (E0), E8, E19, and egg weight loss between E0 and E19 (LSmeans ± SEM) (Experiment 2).

	Egg wt E0, g	Egg wt E8, g	Egg wt E19, g	Egg wt loss E0 – E19, %
N	1,949	1,912	1,535	1,535
Minerals source				
Inorganic	61.39	58.75	55.35	9.83
Organic	61.28	58.59	55.01	10.18
SEM	0.03	0.04	0.06	0.08
Translucency score				
1 + 2 + 3	61.32	58.73	55.34	9.69
5	61.35	58.61	55.02	10.33
SEM	0.03	0.04	0.06	0.08
Interaction				
Inorganic × 1 + 2 + 3	61.43^a^	58.88^a^	55.67^a^	9.39^c^
Inorganic × 5	61.34^ab^	58.62^b^	55.04^b^	10.27^ab^
Organic × 1 + 2 + 3	61.21^b^	58.57^b^	55.02^b^	9.98^b^
Organic × 5	61.36^ab^	58.61^b^	55.01^b^	10.38^a^
SEM	0.04	0.05	0.09	0.10
*P*-values				
Minerals source	0.03	0.003	<0.001	0.002
Translucency score	0.55	0.04	<0.001	<0.001
Interaction	0.01	0.006	<0.001	0.02

a,b LSmeans within a column and factor lacking a common superscript differ (*P* ≤ 0.05).

Fertility was 98.2% on average and not affected by trace minerals source in the broiler breeder diet (*P* = 0.48), translucency score (*P* = 0.59) or the interaction between these 2 (*P* = 0.19). The percentage of vital embryos at E19 (as percent of fertile eggs) was on average 94.1% and not affected by trace minerals source in the broiler breeder diet (*P* = 0.13), translucency score (P = 0.66) or the interaction between these 2 (*P* = 0.86). Hatchability of the eggs transferred at E19 was on average for all treatments 98.5%.

### Egg Composition

#### Experiment 1

Yolk weight, albumen weight, shell weight, or eggshell thickness were neither affected by trace minerals source in the broiler breeder diet (all *P* > 0.29), nor translucency score (all *P* > 0.43), nor the interaction between trace minerals source and translucency score (all *P* > 0.44; Table 4).

**Table 4:** Effects of trace minerals (Zn, Mn, Cu, Se) source (inorganic vs organic) in the broiler breeder diet (55 to 57 wk of age) and egg translucency score (1 + 2 vs. 5) on egg composition and bacterial load on the eggshell before incubation (LSmeans ± SEM) (N = 20 per trace minerals source per translucency score; Experiment 1).

	Egg wt, g	Yolk wt, g	Albumen wt, g	Shell wt, g	Eggshell thickness, mm	Bacterial load, CFU/egg
Minerals source						
Inorganic	66.29	21.62	38.73	5.93	0.365	26
Organic	66.22	21.71	38.62	5.94	0.358	26
SEM	0.15	0.22	0.22	0.07	0.005	5
Translucency score						
1 + 2	66.23	21.75	38.55	5.93	0.361	19
5	66.29	21.59	38.81	5.94	0.362	33
SEM	0.15	0.22	0.22	0.07	0.005	5
Interaction						
Inorganic × 1 + 2	66.13	21.59	38.64	5.90	0.366	16
Inorganic × 5	66.45	21.67	38.83	5.95	0.365	36
Organic × 1 + 2	66.33	21.91	38.46	5.96	0.366	22
Organic × 5	66.13	21.50	38.79	5.93	0.359	30
SEM	0.22	0.31	0.32	0.10	0.007	7
*P*-values						
Minerals source	0.78	0.81	0.73	0.87	0.29	0.93
Translucency score	0.79	0.62	0.43	0.93	0.89	0.34
Interaction	0.25	0.44	0.84	0.74	0.86	0.02^1^

1 Effect disappeared after correction for Bonferroni.

#### Experiment 2

An interaction between trace minerals source in the broiler breeder diet and translucency score was found for albumen weight (*P* = 0.03), but after correction for Bonferroni this effect disappeared. No further interactions or main effects were found on egg composition (all *P* > 0.11; Table 5).

**Table 5:** Effects of trace minerals (Zn, Mn, Cu, Se) source (inorganic vs. organic) in the broiler breeder diet (34 to 36 wk of age) and egg translucency score (1 + 2 + 3 vs. 5) on egg composition and bacterial load on the eggshell before incubation (LSmeans ± SEM) (N = 20 per trace minerals source per translucency score; Experiment 2).

	Egg wt, g	Yolk wt, g	Albumen wt, g	Shell wt, g	Eggshell thickness, mm	Bacterial load, CFU
Minerals source						
Inorganic	61.02	18.49	37.13	5.42	0.366	124^b^
Organic	61.14	18.42	37.36	5.38	0.360	154^a^
SEM	0.14	0.13	0.15	0.05	0.004	29
Translucency score						
1 + 2 + 3	61.09	18.54	27.16	5.41	0.364	175^a^
5	61.06	18.36	37.32	5.40	0.362	103^b^
SEM	0.14	0.13	0.15	0.05	0.004	29
Interaction						
Inorganic × 1 + 2 + 3	60.92	18.63	36.80	5.48	0.371	180
Inorganic × 5	61.12	18.34	37.45	5.37	0.361	68
Organic × 1 + 2 + 3	61.27	18.45	37.51	5.34	0.357	170
Organic × 5	1.01	18.38	37.20	5.43	0.363	139
SEM	0.20	0.19	0.21	0.08	0.005	42
*P*-values						
Minerals source	0.57	0.72	0.29	0.60	0.24	0.004
Translucency score	0.89	0.35	0.44	0.82	0.70	0.02
Interaction	0.27	0.56	0.03^1^	0.20	0.11	0.30

a,b LSmeans within a column and factor lacking a common superscript differ (*P* ≤ 0.05).

1 Effect disappeared after correction for Bonferroni.

### Bacterial Load on the Eggshell

#### Experiment 1

An interaction between trace minerals source in the breeder diet and translucency score (*P* = 0.02) was found, but after correction for Bonferroni this effect disappeared (Table 4).

#### Experiment 2

Eggs obtained from organic trace minerals fed breeders had more CFU on the eggshell than eggs obtained from inorganic trace minerals fed breeders (Δ = 30, *P* = 0.004). Eggs with a low translucency score had more CFU on their eggshells than eggs with a high translucency score (Δ = 72, *P* = 0.02; Table 5).

### Embryonic Tibia Ossification

#### Experiment 1

**Figure 2:**
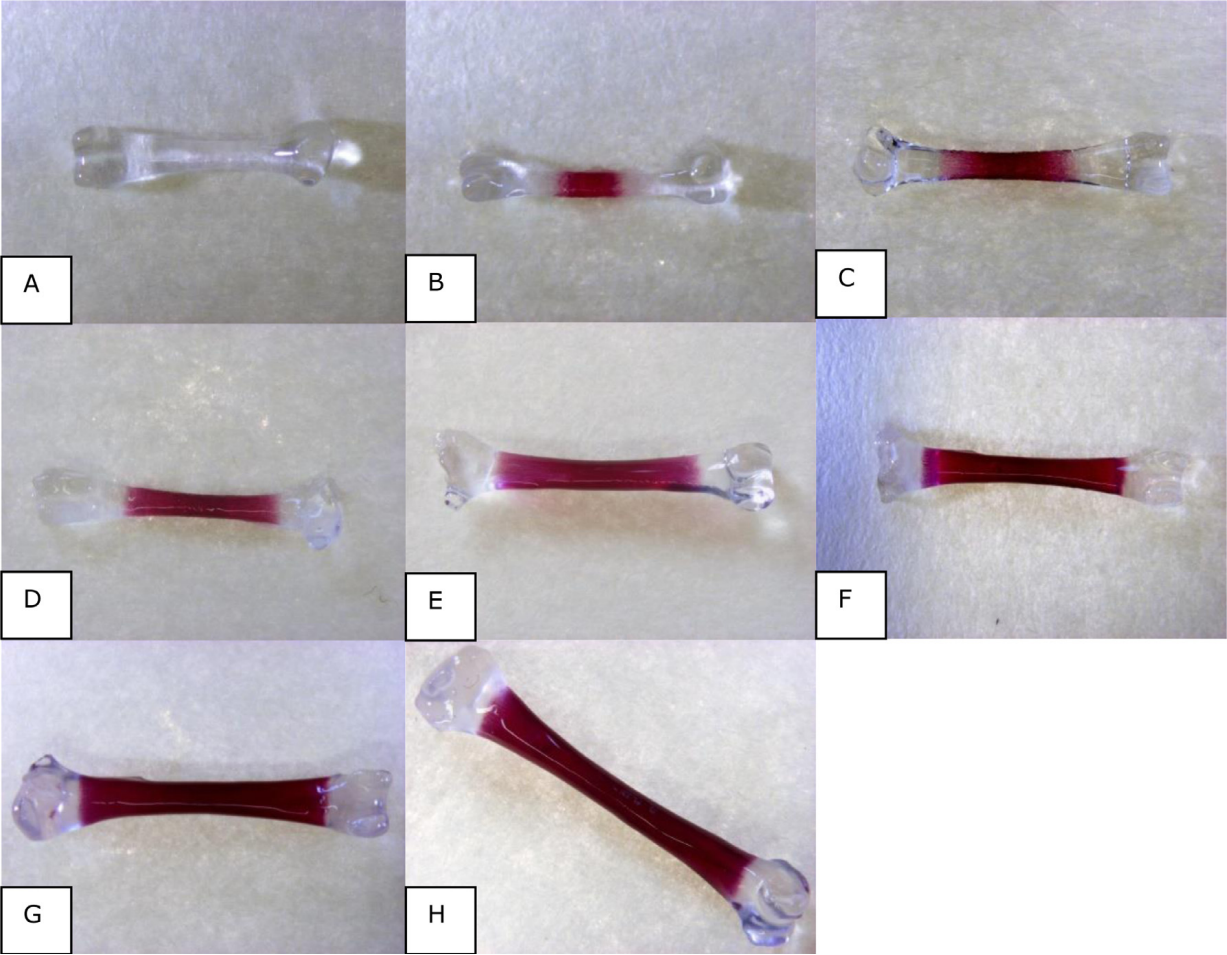
Examples of ossification in tibia at day E8.5 (without ossification; A), at E8.5 (with ossification; B), E9.5 (C), E10.5 (D), E11.5 (E), E12.5 (F), E13.5 (G), and E14.5 (H).

At E8.5, some tibias did not show any ossification (Figure 2A), whereas other days already showed ossification (Figure 2B). Tibia length, ossified length, and ossification percentage did not show an interaction between trace minerals source and egg translucency score, nor a main effect of trace minerals source or translucency score at any of the sampling days was found (E 8.5–E 14.5; Figures 2C–2H; data not shown).

#### Experiment 2

Tibia length, ossified length, nor ossification percentage showed an interaction between trace minerals source and translucency score at any of the sampling days and neither an effect of trace minerals source was found at any of the sampling days (data not shown). At E11.5, embryos in eggs with a low translucency score had more tibia ossified than in eggs with a high translucency score (6.78 vs. 6.53 mm; *P* = 0.03), whereas the opposite was found at E13.5 (11.39 vs. 11.73 mm; *P* = 0.05). For the other days, no effect of translucency score was found.

### Embryonic Heat Production

#### Experiment 1

**Figure 3:**
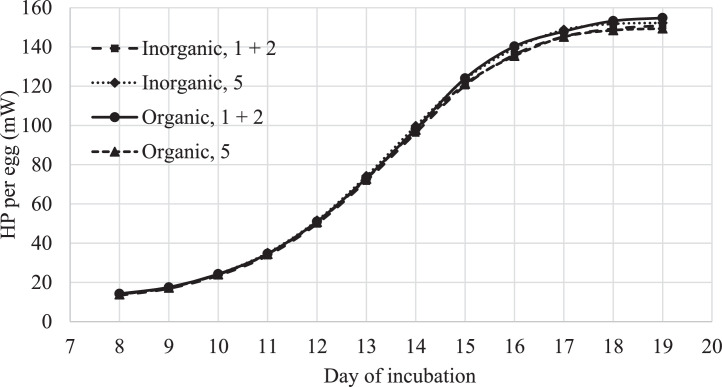
Effects of trace minerals (Zn, Mn, Cu, Se) source (inorganic vs organic) in the broiler breeder diet (55 to 57 wk of age) and egg translucency score (1 + 2 vs. 5) on embryonic heat production (HP) from E8 to E19 (LSmeans) (N = 2 per trace minerals source per translucency score; Experiment 1).

Average embryonic heat production between E8 and E19 was 85.1 mW per egg and was not affected by trace minerals source (*P* = 0.84), translucency score (*P* = 0.74) or the interaction between these two factors (*P* = 0.27; Figure 3). RQ was on average 0.75 and was not affected by trace minerals source (*P* = 0.74), translucency score (*P* = 0.61) or the interaction between these 2 factors (*P* = 0.63; data not shown).

#### Experiment 2

Average embryonic heat production between E8 and E19 was 81.0 mW per egg and was not affected by trace minerals source *(P* = 0.84), translucency score (*P* = 0.51), or the interaction between these 2 factors (*P* = 0.95; data not shown). RQ was on average 0.76 and was also not affected by trace minerals source (*P* = 0.53), translucency score (*P* = 0.10) or the interaction between these 2 factors (*P* = 0.77; data not shown).

### Hatching Characteristics

#### Experiment 1

An interaction between trace minerals source in the breeder diet and translucency score was found for heart percentage (*P* = 0.02) and liver percentage (*P* = 0.009; Table 6). The effect for heart percentage disappeared after correction for Bonferroni. Liver percentage was not affected by trace minerals source in low translucent eggs, but in high translucent eggs, organic trace minerals in the breeder diet resulted in higher liver percentage than inorganic trace minerals (Δ = 0.14%). Residual yolk weight (Δ = 0.42 g; *P* = 0.04) was lower and intestine percentage (Δ = 0.22%; *P* = 0.04) was higher in chickens originating from organic trace minerals fed breeders compared to chickens originating from inorganic trace minerals fed breeders.

**Table 6:** Effects of trace minerals (Zn, Mn, Cu, Se) source (inorganic vs. organic) in the broiler breeder diet (55 to 57 wk of age) and egg translucency score (1 + 2 vs. 5) on chicken characteristics at 6 h after hatching (LSmeans ± SEM) (N = 33 or 34 per trace minerals source per translucency score; Experiment 1).

	Hatch time, h	Chicken wt, g	Navel score^1^	Hock score^1^	Beak score^1^	YFBM, g^2^	RY, g^2^	Heart wt, % YFBM	Liver wt, % YFBM	Stomach wt, % YFBM	Intestine wt, % YFBM
Minerals source											
Inorganic	502	49.05	0.57	0.30	0.12	41.68	7.37^a^	0.80	2.36	5.97	4.80^b^
Organic	502	48.88	0.39	0.24	0.18	41.93	6.95^b^	0.81	2.41	5.94	5.02^a^
SEM	1	0.19	-	-	-	0.20	0.19	0.02	0.03	0.10	0.09
Translucency score											
1 + 2	502	48.96	0.46	0.22	0.18	41.73	7.23	0.80	2.39	5.90	4.93
5	502	48.97	0.50	0.32	0.12	41.88	7.09	0.82	2.39	6.01	4.89
SEM	1	0.19	-	-	-	0.20	0.19	0.02	0.03	0.10	0.09
Interaction											
Inorganic × 1 + 2	502	49.07	0.53	0.21	0.09	41.69	7.39	0.82	2.41^ab^	5.93	4.80
Inorganic × 5	502	49.03	0.61	0.39	0.15	41.67	7.36	0.78	2.32^b^	6.00	4.80
Organic × 1 + 2	502	48.84	0.38	0.24	0.26	41.77	7.08	0.79	2.36^ab^	5.86	5.05
Organic × 5	502	48.91	0.39	0.24	0.09	42.09	6.82	0.84	2.46^a^	6.01	4.97
SEM	1	0.24	-	-	-	0.25	0.24	0.02	0.04	0.13	0.12
*P*-values											
Minerals source	0.89	0.40	0.14	0.43	0.58	0.25	0.04	0.49	0.16	0.78	0.04
Translucency score	0.62	0.98	0.44	0.07	0.98	0.50	0.50	0.56	1.00	0.35	0.68
Interaction	1.00	0.81	0.83	0.22	0.16	0.45	0.59	0.02^3^	0.009	0.72	0.68

a,b LSmeans within a column and factor lacking a common superscript differ (*P* ≤ 0.05).

1 Navel scored as 0 = clean, 1 = small button or strain or 2 = large button; Hock scored as 0 = no discoloration, 1 = red discoloration; Beak scored as 0 = no discoloration, 1 = red discoloration.

2 YFBM = yolk-free body mass; RY = residual yolk.

3 Effect disappeared after correction for Bonferroni.

No interaction between trace minerals source and translucency score (all *P* > 0.15), nor an effect of trace minerals source (all *P* > 0.24) or translucency score (all *P* > 0.41) was found on tibia characteristics at 6 h after hatching (Table 8).

#### Experiment 2

An interaction between trace minerals source and translucency score was found for hatch time (*P* < 0.001). In inorganic trace minerals fed breeders, no effect of translucency score on hatch time was found, but in organic trace minerals fed breeders, eggs with a low translucency score hatched earlier than eggs with a high translucency score (Δ = 10 h; Table 7). Chickens originating from organic trace minerals fed breeders showed more red hocks (Δ = 0.15; *P* = 0.05) and had lower stomach percentage (Δ = 0.21%; *P* = 0.04) than chickens originating from inorganic trace minerals fed breeders. A low translucency score resulted in chickens with a higher RY weight (Δ = 0.35 g; *P* = 0.04), lower heart percentage (Δ = 0.12%; *P* < 0.001) and lower liver percentage (Δ = 0.19%; *P* < 0.001) than a high translucency score. After correction for hatch time these effects disappeared.

**Table 7:** Effects of trace minerals (Zn, Mn, Cu, Se) source (inorganic vs organic) in the broiler breeder diet (34 to 36 wk of age) and egg translucency score (1 + 2 + 3 vs 5) on chicken characteristics at 6 h after hatching (LSmeans ± SEM) (N = 33 or 34 per minerals source per translucency score; Experiment 2).

	Hatch time, h	Chicken wt, g	Navel score^1^	Hock score^1^	Beak score^1^	YFBM, g^2^	RY, g^2^	Heart wt, % YFBM	Liver wt, % YFBM	Stomach wt, % YFBM	Intestine wt, % YFBM
Minerals source											
Inorganic	499	45.31	0.31	0.18^b^	0.22	39.31	6.00	0.82	2.44	6.12^a^	4.78
Organic	500	45.40	0.47	0.33^a^	0.15	39.19	6.22	0.82	2.42	5.91^b^	4.75
SEM	1	0.13	-	-	-	0.12	0.11	0.01	0.03	0.07	0.07
Translucency score											
1 + 2 + 3	497	45.50	0.39	0.30	0.22	39.23	6.29^a^	0.76^b^	2.33^b^	5.99	4.70
5	502	45.21	0.39	0.21	0.15	39.27	5.94^b^	0.88^a^	2.52^a^	6.04	4.84
SEM	1	0.13	-	-	-	0.12	0.12	0.01	0.03	0.07	0.07
Interaction											
Inorganic × 1 + 2 + 3	498^b^	45.40	0.29	0.24	0.24	39.33	6.07	0.76	0.36	6.12	4.75
Inorganic × 5	499^b^	45.22	0.32	0.12	0.21	39.29	5.93	0.88	2.52	6.13	4.81
Organic × 1 + 2 + 3	495^b^	45.60	0.48	0.36	0.21	39.13	6.50	0.76	2.30	5.87	4.64
Organic × 5	505^a^	45.20	0.45	0.30	0.09	39.26	5.94	0.88	2.53	5.95	4.87
SEM	1	0.19	-	-	-	0.17	0.16	0.02	0.04	0.10	0.10
*P*-values											
Minerals source	0.62	0.65	0.09	0.05	0.24	0.51	0.18	0.93	0.64	0.04	0.76
Translucency score	<0.001	0.13	0.74	0.28	0.28	0.81	0.04	<0.001	<0.001	0.67	0.16
Interaction	<0.001	0.56	0.74	0.58	0.36	0.64	0.20	0.92	0.33	0.72	0.42

a,b LSmeans within a column and factor lacking a common superscript differ (*P* ≤ 0.05).

1 Navel scored as 0 = clean, 1 = small button or strain or 2 = large button; Hock scored as 0 = no discoloration, 1 = red discoloration; Beak scored as 0 = no discoloration, 1 = red discoloration.

2 YFBM = yolk free body mass; RY = residual yolk.

**Table 8:** Effects of trace minerals (Zn, Mn, Cu, Se) source (inorganic vs. organic) in the broiler breeder diet (55 to 57 wk) and egg translucency score (1 + 2 vs. 5) on chicken tibia characteristics at 6 h after hatching (LSmeans ± SEM) (N = 10 per minerals source per translucency score; Experiment 1).

	Tibia length, mm	Ossified length, mm	Diaphysis thickness, mm	Tibia volume, mm^3^	Ossified volume, mm^3^	Ossified mass, mg	Bone mineral density, mg/mm^3^	Marrow volume, mm^3^
Minerals source								
Inorganic	34.1	26.7	1.82	435	45.0	81.2	1.81	108
Organic	34.0	26.8	1.84	444	46.3	83.6	1.81	108
SEM	0.1	0.1	0.02	12	0.8	1.4	0.01	2
Translucency score								
1 + 2	34.1	26.6	1.84	438	45.7	82.7	1.81	107
5	34.1	26.8	1.82	431	45.5	82.2	1.81	108
SEM	0.1	0.1	0.02	12	0.8	1.4	0.01	2
Interaction								
Inorganic × 1 + 2	34.2	26.7	1.84	431	45.5	82.3	1.81	107
Inorganic × 5	34.0	26.7	1.81	440	44.4	80.2	1.81	108
Organic × 1 + 2	34.0	26.6	1.84	451	45.9	83.0	1.81	108
Organic × 5	34.1	26.9	1.84	437	46.6	84.2	1.80	108
SEM	0.1	0.2	0.03	17	1.1	1.9	0.01	3
*P*-values								
Minerals source	0.68	0.65	0.49	0.63	0.25	0.24	0.94	1.00
Translucency score	0.80	0.41	0.50	0.87	0.90	0.81	0.43	0.86
Interaction	0.15	0.41	0.57	0.50	0.41	0.41	0.98	0.91

No interaction between trace minerals source and translucency score (all *P* > 0.15), nor an effect of trace minerals source (all *P* > 0.10), or translucency score (all *P* > 0.16) was found on tibia characteristics at 6 h after hatching (Table 9).

**Table 9:** Effects of trace minerals (Zn, Mn, Cu, Se) source (inorganic vs. organic) in the broiler breeder diet (34 to 36 wk of age) and egg translucency score (1 + 2 + 3 vs. 5) on chicken tibia characteristics at 6 h after hatching (LSmeans ± SEM) (N = 10 per minerals source per translucency score; Experiment 2).

	Tibia length, mm	Ossified length, mm	Diaphysis thickness, mm	Tibia volume, mm^3^	Ossified volume, mm^3^	Ossified mass, mg	Bone mineral density, mg/mm^3^	Marrow volume, mm^3^
Minerals source								
Inorganic	33.6	26.0	1.71	374	39.5	70.5	1.79	101
Organic	33.6	25.9	1.77	353	39.7	70.6	1.78	97
SEM	0.4	0.1	0.04	9	0.7	1.2	0.01	2
Translucency score								
1 + 2 + 3	33.6	26.1	1.77	365	39.7	70.9	1.79	97
5	33.6	5.8	1.71	362	39.5	70.3	1.78	100
SEM	0.4	0.1	0.04	9	0.7	1.2	0.01	2
Interaction								
Inorganic × 1 + 2 + 3	33.6	26.3	1.76	379	39.9	71.3	1.79	100
Inorganic × 5	33.6	25.7	1.67	370	39.1	69.7	1.78	101
Organic × 1 + 2 + 3	33.6	25.8	1.78	352	39.5	70.6	1.79	95
Organic × 5	33.7	25.9	1.75	354	39.8	70.9	1.78	100
SEM	0.5	0.2	0.05	12	1.0	1.7	0.01	3
*P*-values								
Minerals source	0.99	0.48	0.35	0.10	0.85	0.87	0.91	0.22
Translucency score	1.00	0.16	0.32	0.79	0.84	0.71	0.50	0.26
Interaction	0.87	0.07	0.61	0.67	0.56	0.58	0.81	0.50

## DISCUSSION

This study aimed to investigate effects of trace minerals (Zn, Mn, Cu, Se) source and egg translucency on egg characteristics, embryo development, and hatchling quality in both old and prime flock broiler breeders. The hypothesis of this study was that organic trace minerals in the breeder diet would be transferred into the egg, resulting in positive effects on eggshell quality and embryonic bone development in the offspring. Results showed that trace minerals source and translucency interacted for egg weight (loss) during incubation, but effects on tibia characteristics throughout incubation and at hatch and effects on hatchling characteristics were limited. Results will be discussed per main effect, where the above mentioned interaction effects will be included in the trace minerals source section.

### Trace Mineral Source

Organic minerals in general have been shown to have a higher bioavailability than inorganic minerals (for review see Torres and Korver, 2018), which might result in a higher transfer of minerals from the breeder to the egg and consequently to the offspring (Favero et al., 2013a; Xiao et al., 2015; Zhu et al., 2015). In the current study, replacement of inorganic trace minerals (Zn, Mn, Cu, Se) by their organic varieties affected particularly egg weight loss during incubation, without affecting eggshell weight and thickness. In other studies about organic trace minerals in broiler breeders, effects on eggshell characteristics (thickness, weight, breaking strength) were ambiguous. Favero et al. (2013b), Araújo et al. (2019) and Yacoob et al. (2020) found positive effects of organic trace minerals on eggshell characteristics compared to their inorganic varieties, whereas no effect was found by Urso et al. (2015) and Wang et al. (2019b) and in the current study. Differences among studies might be related to the composition of the mixture of trace minerals that were replaced from their inorganic varieties to their organic varieties, or the concentration of the trace minerals, or the type of organic minerals (amino acid, protein). Egg weight loss (and consequently eggshell conductance) was higher in eggs from organic trace minerals fed breeders, especially in eggs with a low translucency as expressed by the interaction between trace minerals source and translucency score. It appears that trace minerals bound to an amino acid fed to broiler breeders affects the ultrastructure of the eggshell or egg membranes, resulting in a higher conductance especially in eggs with a low translucency score. However, whether or not this is related to the structure of the palisade layer, the mammillary layer or the membranes remains unclear. The idea that organic trace minerals particularly affect the eggshell ultrastructure (Xiao et al., 2014; Zhang et al., 2017) is supported by the finding that in the experiment 2 (with a quite high bacterial load), eggs obtained from the organic trace minerals fed breeders had a higher bacterial load on the eggshell than the eggs obtained from the inorganic trace minerals fed breeders. It was hypothesized that in the current study due to the warm PBS used, the bacteria are sucked out of the eggshell and with a more open ultrastructure more bacteria can hide in the eggshell and consequently obtained bacterial load per egg was higher. In the old breeder flock (experiment 1), with a much lower bacterial load, no effects of trace minerals source were found. Another finding that supports our hypothesis that organic minerals may affect the ultrastructure of the eggshell and consequently the eggshell conductance is the lower RY (experiment 1) in hatchlings originating from organic trace minerals fed breeders compared to the hatchlings originating from inorganic trace minerals fed breeders. With a higher conductance, due to a more open eggshell ultrastructure, more oxygen can enter the egg and consequently, more nutrients from the yolk can be oxidized by the embryo (Nangsuay et al., 2017). This could partially explain the higher organ weights in embryos from hens fed organic trace minerals in experiment 1.

In the current study, no effect of trace minerals source on fertility was found, which is supported by other studies (Favero et al., 2013b; Zhu et al., 2015; Araújo et al., 2019; Wang et al., 2019b). Effects of trace minerals source on chicken quality at hatch were largely absent in the current study, which is supported by other studies (Favero et al., 2013b; Urso et al., 2015; Zhu et al., 2015). In contrast to Favero et al (2013a), in the current study no effects of trace minerals source in the breeder diet were found on embryonic tibia ossification. Favero et al. (2013a) found that organic trace minerals in the Cobb 500 breeder diet (Zn, Mn, Cu) stimulated tibia ossification at E14 and E18 and increased the moment of inertia of tibia bone at hatch, although no effects of trace minerals source was found on tibia characteristics at E10 and hatch, which support findings in the current study.

### Egg Translucency

Egg translucency score was considerably higher in the prime flock compared to the old flock, which is not in accordance with Roberts et al. (2013), who demonstrated an increase in egg translucency in older flocks (55–65 wk of age) compared to flocks of 25 to 40 wk or 40 to 55 wk of age. However, in the latter study, flocks of >65 wk of age showed comparable translucency scores than the younger flocks. Baker and Curtis (1957) and Zhang et al. (2021) showed that egg translucency differed between white leghorn strains and Baker and Curtis (1957) showed that egg translucency largely varied between individual hens, and eggs of the same hen showed less variability in translucency. This all suggests that egg translucency appears to be strongly determined by individual hen or flock characteristics, rather than flock age. Wang et al. (2017) showed that eggs with a high translucency score had thicker eggshells and thinner shell membranes than eggs with a low translucency score. In the current study, this could not be confirmed, but the large difference found in egg translucency between flocks of the same strain (Ross 308), suggests that other factors than genetics play a large role in egg translucency. Whether or not diet is playing a role on egg translucency is not clear.

In the current study, no effect of egg translucency was found on egg composition (albumen, yolk), which is in accordance with some old studies (Baker and Curtiss, 1957, 1958), but Wang et al. (2017) found higher eggshell weights and eggshell thickness in high translucent eggs compared to less translucent eggs, which was not found in the current study. Nevertheless, in the current study, high translucent eggs had a higher egg weight loss during incubation than low translucent eggs, suggesting that the eggshell conductance was higher and thus differences in eggshell and/or eggshell membrane quality (e.g., mammillary layer quality, eggshell membrane thickness or pore branching; Chousalkar et al., 2010; Ray et al., 2015; Wang et al., 2017) were present between high and low translucent eggs, which were not expressed in differences in eggshell thickness or eggshell weight. The lack of differences in albumen and yolk weights between eggs with different translucency score was also expressed in the lack of effects on metabolic heat production and respiration quotient. Yolk weight is strongly related to energy used and consequently to metabolic heat production (Nangsuay et al., 2013).

Chousalkar et al. (2010) found a positive correlation between egg translucency score and egg shell penetration by *Salmonella* and *E. coli*. This appears to contradict to the findings in the current study, where no effect of egg translucency on bacterial load on the eggshell was found in experiment 1, but a lower bacterial load on the eggshell was found in high translucent eggs than in low translucent eggs in experiment 2. In the current study, no differentiation in bacterial species was made and also the method of bacterial collection differed with the study of Chousalkar et al. (2010). In the latter study, they swapped the eggshells, whereas in the current study, eggs were rolled in warm water, aiming to remove all bacteria also from inside the eggshell pores. Eggs with a high egg translucency score have more externally branching pores than eggs with a low egg translucency score (Ray et al., 2015), and it can be speculated that it is more difficult to remove bacteria from branched pores than from straight pores (in eggs with a low egg translucency score), resulting in lower CFU.

In experiment 2, high translucent eggs resulted in a lower RY weight and higher relative heart and liver weights. These finding might be related to the later hatching of the chickens originating from eggs with a high translucency score compared to low translucency score (Δ = 7 h). When these variables were corrected for hatching time, differences in these chicken characteristics between translucency scores disappeared. The reason that in experiment 2 high translucent eggs hatched earlier (particularly from the organic trace minerals fed breeders) than the low translucent eggs remains unclear. It also remains unclear why this difference was not found in experiment 1 (old breeder flock). It can be hypothesized that this discrepancy is related to the general higher eggshell conductance in older flocks (Peebles and Brake, 1987; de Araújo et al., 2017) and consequently, egg translucency in old flocks is playing a smaller role in egg weight loss and consequently hatching time. The lack of effects on tibia ossification during incubation and mineralization at hatching suggests that egg translucency does not affect the amount and speed of Ca and/or P uptake from the eggshell.

It can be concluded that organic trace minerals in broiler breeder diets affect eggshell conductance, particularly in low translucent eggs. Effects of trace minerals source in the broiler breeder diet on hatchling quality in general and tibia characteristics in particular appears to be limited. Egg translucency appears to be strongly determined by individual flock characteristics, rather than breeder age, and affects eggshell conductance and some hatchling characteristics.
